# Procuring family planning methods for every woman in the world

**DOI:** 10.1186/1742-4755-9-21

**Published:** 2012-09-15

**Authors:** Rosie Smith, José M Belizán

**Affiliations:** 1 BioMed Central, 236 Gray's Inn Road, London, WC1X 8HB, UK; 2 Institute for Clinical Effectiveness and Health Policy (IECS), Dr Emilio Ravignani 2024, Buenos Aires, C1414CPV, Argentina

## 

Every year the lack of access to contraceptives leads to 60 million unwanted pregnancies, 22 million unsafe abortions and 3 million infant deaths [1,2], the majority of which occur in developing countries. Women all around the globe are unable to choose when they fall pregnant, nor control the size of their family, because contraception is simply not accessible. Reducing fertility would not only prevent 358,000 [2] maternal deaths each year, but would also significantly contribute to eradicating extreme poverty, promoting the empowerment of women and ensuring environmental and economic sustainability. Initiatives like Every Woman, Every Child [3] and the 5^th^ Millennium Development Goal [4] advocate that wider access to contraception in developing nations would provide women more opportunity to work, learn new skills and generate income.

In July this year the landmark Family Planning Summit was held in London, aiming to raise awareness of the 222 million women in desperate need of contraception [1] and begin steps to securing the financial and political commitment towards solving this problem. Held jointly between the UK Government and The Bill & Melinda Gates Foundation, the summit brought together international powerhouses including national governments, non-governmental organisations and private sector companies aiming to break down the barriers to contraception. The summit provided a stage for governments such as Ethiopia, Senegal and Nigeria to demonstrate their commitment to family planning and allowed the heads of state from Tanzania, Rwanda, Malawi amongst others, to publically highlight their successes in disseminating contraception to their population, and outline their goals further spread this critical component of reproductive health throughout their nations. In total $2.6 billion was pledged at the Summit [5] which will be used to improve supply chains to contraception, aid countries to reach their family planning goals, and encourage the development of dynamic family planning programs. However, overarching the commitments and goals of individual nations, the Family Planning Summit will ensure that access to contraception is a fundamental right for women of generations to come.

With the population of the world growing at a rate never seen before, social and economic benefits of contraception have never been so imperative. At the household level, long term follow-up from the family planning Matlab study in Bangladesh showed an increase in both mother and child’s body mass index, as well as enhanced primary education and increased earnings for mothers [6]. Such benefits of contraception can have a direct effect on the positive growth of a community - decreasing youth dependency and increasing the working aged population [7,8]. Family planning also enables girls who may be underdeveloped physically or emotionally immature to delay their first pregnancy, allowing them to enjoy a complete childhood before experiencing motherhood. With 14.3 million births to adolescents in 2008 [9], 91% occurring in developing countries, 15–19 year olds are particularly vulnerable to risks such as unsafe abortions with 1 in 4 unsafe abortions in Africa occurring on an adolescents [10].

One of the most significant repercussions of family planning is the direct effect on maternal and neonatal mortality. Increased contraception has not only reduced the maternal mortality ratio by 26% in the past 10 years but it is projected that another 30% of maternal deaths [8] would be prevented by fulfillment of the unmet need for contraception. By reducing the number of pregnancies, complications associated with parturition are naturally reduced, in turn diminishing death through unsafe abortion practices and the associated dangers of high parities. Evidence has shown by allowing women to space their pregnancies the risk of premature birth or high risk pregnancies is lowered and child survival rates are greatly increased [11-14].

Overall the Family Planning Summit is a positive step towards the empowerment of women and the bridging of the gender equality gap. It will provide access to resources and information to allow all women to take control of the size and timings of their family. *Reproductive Health* wholeheartedly supports the Family Planning initiative, and would like to contribute to this important movement through the publication of research concerning contraception and its wide-reaching effects.
